# Effects of Chemicals Exposure on the Durability of Geopolymer Concrete Incorporated with Silica Fumes and Nano-Sized Silica at Varying Curing Temperatures

**DOI:** 10.3390/ma16186332

**Published:** 2023-09-21

**Authors:** Sagar Paruthi, Ibadur Rahman, Asif Husain, Mohd Abul Hasan, Afzal Husain Khan

**Affiliations:** 1 Department of Civil Engineering, Jamia Millia Islamia, New Delhi 110025, India; irahman1@jmi.ac.in (I.R.); asifjmi@gmail.com (A.H.); 2 Civil Engineering Department, College of Engineering, King Khalid University, Abha 61421, Saudi Arabia; mohad@kku.edu.sa; 3 Department of Civil Engineering, College of Engineering, Jazan University, P.O. Box 706, Jazan 45142, Saudi Arabia; amuafzal@gmail.com

**Keywords:** nano-silica, alccofine, durability, geopolymer concrete, silica fumes

## Abstract

Durable concrete significantly reduces the spalling caused by chemical damage. The objective of current research is to substitute cement with supplementary such as fly ash (FA), ground granulated blast furnace slag (GGBS), and alccofine (AF). Additionally, the impact of nano-silica (NS) and silica fumes (SF) on the GPC durability when cured at various temperatures has been attempted. In order to perform this, GPC samples were produced by combining NS and SF at proportions of 0.5% NS + 5% SF, 1% NS + 10% SF, and 1.5% NS + 15% SF, and then cured at temperatures of 27 °C, 60 °C, 90 °C, and 120 °C, respectively. In this research, all concrete specimens were continuously immersed for twelve weeks under four different chemicals, i.e., HCl (2%), H_2_SO_4_ (2%), NaCl (6%), and Na_2_SO_4_ (6%). The influence of chemical attack on the qualities of concrete was examined by evaluating the water absorption, sorptivity, loss of mass, and loss of GPC strength. The durability aspect is also studied by visual appearance and mass loss under harmful chemical attack. The combination of GPC with integrated NS and SF affords great resistance against chemical attacks. The percentages of these two components are 1.5% and 15%. For GPC specimens, when cured at 90 °C, the resultant strength is found at its maximum.

## 1. Introduction

Construction material used in large amounts nowadays is cement concrete, and concrete deterioration due to sulfate and chloride attacks is very much observed worldwide [1,2,3,4,5]. After the sulfate attack, the permeability and porosity of the concrete surface are increased due to the release of hydroxide and calcium ions from the matrix. This ends up in deteriorated concrete [6]. Concrete deteriorates after the chemical reactions that take place between the harmful acidic environment and the calcium present in concrete [7]. Cement production is the leading cause of the release of half of the harmful gases in the atmosphere, mainly CO_2_ [8]. To reduce the excess release of CO_2_, new greener material, i.e., alkali-activated geo-polymerised concrete, is introduced in the industry of construction worldwide [9]. This type of green concrete is manufactured after alkaline-activated source materials, which have binding properties as well, such as FA, GGBFS, volcanic pumice dust (VPD), palm oil fuel ash (POFA), and quarry dust [10,11,12,13,14,15]. Its workability is less as an alkaline solution is added, which is a viscous basic solution, so it is important to add a superplasticiser to improve its workability [16,17]. Geopolymerisation is responsible for the strength of GPC; on the other hand, the heat of hydration is responsible for strength in conventional concrete (CC) [18,19]. Using GPC in construction also helps solve the problem of disposing of different kinds of industrial waste [20,21]. The movement of ions into the concrete after exposing the surface to harmful chemicals results in deteriorated concrete, and GPC proves to be a better material than CC in terms of durability [22]. Many researchers observed that GPC has low penetrability of chloride ions and high resistance to the sulfate attack as well [23,24,25]. The durability of GPC is enhanced after partially replacing FA with GGBS [26,27], silica fumes [28,29], and [30,31,32]. SF is a byproduct obtained during the production of silicon metal and is very much used as an admixture due to its pozzolanic property, which ultimately improves the GPC performance [33]. The polymerisation process is increased by the Si–Al phase, and Si content rises after incorporating nano-materials such as NS, SF, nano-clay, nano-alumina, and nano-metakaolin in GPC [34,35,36]. Using air agents during the mixing process makes the GPC light in weight [37]. The porosity of GPC is very much reduced after replacing the source material with silica fumes (SF) [7,38,39]. The study reveals that the durability of GPC is significantly improved after incorporating NS and SF individually in it [7,33,40,41]. In the examination of sorptivity in GPC infused with SF up to 10%, both under ambient and elevated curing temperatures, a discernible pattern emerged [42]. The inclusion of SF within GPC was associated with a reduction in capillary suction. Interestingly, the introduction of SF did not yield any observable impact on the initial sorption of GPC that underwent high-temperature curing. Furthermore, it was observed that specimens cured under ambient temperatures exhibit notably lower sorptivity rates when compared to those subjected to elevated temperature curing. The incorporation of SF yielded the noteworthy effect of diminishing sorptivity while concurrently enhancing the durability performance of GPC [43]. Fly ash-based GPC exhibits notable chemical resistance and remarkable durability. An intriguing observation was made regarding the strength loss in GPC containing 20% SF following exposure to sulfate and chloride attacks, which was found to be considerably minimal compared to the controlled GPC [7].

Still, the study of the durability of GPC after incorporating NS and SF combination has little literature. The objective of this study is to check the influence of SF and NS addition on (a) GPC properties, particularly in the presence of HCl, H_2_SO_4_, NaCl and Na_2_SO_4_ on GPC specimens, (b) different curing temperatures, and (c) chemical attack on the properties of concrete due to exposure evaluated. To attain the stated objective, three different mix proportions were formulated, namely 0.5% NS + 5% SF, 1% NS + 10% SF, and 1.5% NS + 15% SF, while keeping other parameters constant. The three varied mix samples underwent curing at different temperatures—27 °C, 60 °C, 90 °C, and 120 °C—to investigate the impact of curing temperature on the enduring properties of GPC. Within this investigation, the resilience of GPC infused with NS + SF was evaluated through continuous immersion of GPC specimens in a solution of HCl (2%), H_2_SO_4_ (2%), NaCl (6%), and Na_2_SO_4_ (6%). Subsequent to immersion, assessments were conducted to ascertain water absorption, sorptivity, mass loss and strength reduction.

## 2. Experimental Program

### 2.1. Materials Used in GPC

**Figure 1:**
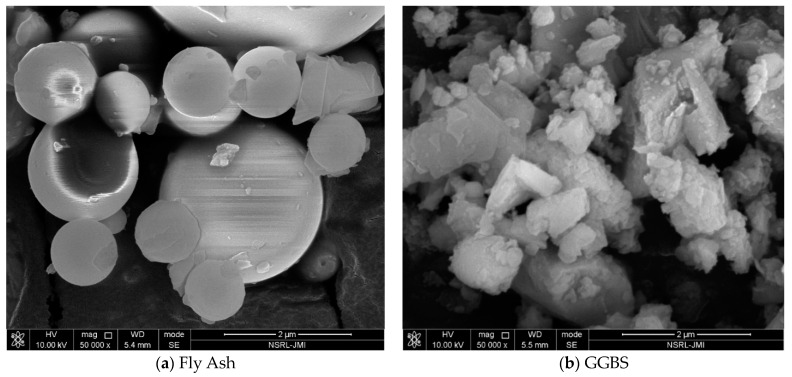

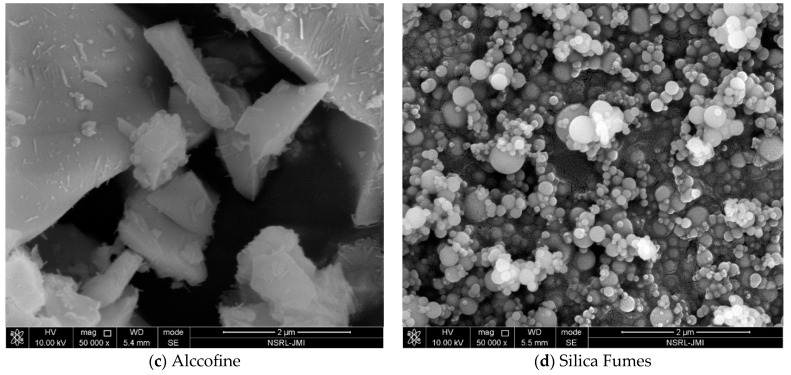
Microstructural images of the (**a**) FA, (**b**) GGBS, (**c**) Alccofine, (**d**) SF.

Three different source materials were used to produce GPC, i.e., FA, GGBS and Alccofine (AF). FA with less calcium content was obtained from N.B. Constructions Private Limited, Rajasthan (India), which satisfies all the specifications in IS 3812:2013 [44]. GGBS was obtained from Prime Cement Private Limited, Behror (India), which meets all the specifications in IS 12089:1987 [45]. SF was obtained from Amorphous Chemicals Private Limited, New Delhi, India, which satisfies all the specifications in IS 15388:2003 [46]. Alccofine (AF) 1203 and NS were obtained from Subhavana Industries, Faridabad (India). Alccofine 1203 is ultrafine slag with low calcium silicate content. The different types of oxides present in FA, GGBS, SF, AF and NS are shown in Table 1. Granite aggregates of grading 7 mm, 10 mm, and 20 mm were used as coarse aggregate (CoAg), confirming the specification of IS 383:1970, and Badarpur sand was used as fine aggregate (FiAg), confirming the specifications of IS 383:1970 [47]. Alkaline solutions were prepared after mixing the NaOH and Na_2_SiO_3_. These two alkaline chemicals were obtained from Babu Ram & Sons Private Limited, Tilak Nagar, New Delhi (India). Sodium silicate is a yellow-colour jelly liquid with a specific gravity of 1.5. Sodium naphthalene formaldehyde-based superplasticiser (SP) in the powdered form was used to increase the workability of GPC, which was obtained from Babu Ram & Sons Private Limited, Tilak Nagar, New Delhi, India. Sodium silicate consists of SiO_2_ (29.29% wt.%), Na_2_O (13.99 wt.%), and water (56.72 wt.%). Table 2 shows the physical characteristics of the material used. The microstructural images of FA, GGBS, AF, and SF are shown in Figure 1.

### 2.2. Design Proportions for Mixture of GPC

In the course of this study, we formulated four distinct variants of geopolymer concrete mixes, denoted as G1A, G2A, G2B, and G2C. Among these, G1A represents the controlled geopolymer concrete mix. On the other hand, G2A, G2B, and G2C were developed by introducing additives—0.5% NS + 5% SF, 1% NS + 10% SF, and 1.5% NS + 15% SF, respectively—while maintaining uniformity in all other parameters. The specific value of these constant parameters is detailed in Table 3. Three precursors were used in this research: FA/GGBS/AF in the ratio of 35% FA:50% GGBS:15% AF. All four types of GPC were activated in a 16 M concentrated NaOH solution. A ratio of Na_2_SiO_3_: NaOH was kept equal to 2.5 for all GPC mixes, and w/b (water:binder) ratio was the same for all GPC mixes. The content of the superplasticiser was kept the same for all GPC mixes. The activator liquid-to-source material ratio was maintained at 0.35, and w/s (water/solid) was observed at 0.16 for all the mixes. 

### 2.3. Methodology

Durability refers to a concrete’s capacity to withstand deterioration over its lifespan, stemming from chemical attack and abrasion. To assess the durability of geopolymer concrete, cubical specimens were meticulously prepared and cured for 28 days under controlled conditions. Subsequently, these specimens were exposed to various chemicals for another 62 days, thereby resulting in an aging period spanning 90 days. This examination considered the impact on compressive strength and weight alterations, compared to controlled GPC cured for 90 days using tap water. The choice of 90-day curing duration was influenced by prior research findings [12,48]. Durability and change in the strength of GPC after adding NS and MS in combination were studied; for this purpose, four different types of the mix proportion were prepared and cast in a cubical mould with 150 mm × 150 mm × 150 mm in size. In this study, four different types of chemicals were used to study the durability of GPC, i.e., hydrochloric acid (HCl), sulphuric acid (H_2_SO_4)_, sodium chloride (NaCl), and sodium sulfate (Na_2_SO_4_). Four tests, i.e., mass loss, water absorption, sorptivity, and compressive strength, were tested for GPC after chemical exposure for 90 days. The concentration of chemicals was maintained by replacing it after every 30 days. All four different mix types were also cured under tape water conditions. All types of GPC mix were cured at 27 °C, 60 °C, 90 °C, and 120 °C for one day in an oven to investigate the durability of GPC cured at different temperatures.

#### 2.3.1. Mass Loss

The casted GPC cube with a size of 150 mm × 150 mm × 150 mm was weighed in dry and wet conditions to study the variation in the GPC weight. The dry weight GPC specimens were determined by keeping the specimens in the open air after one day of heat curing in the oven and weighted on the weighing machine (w_i,dry_). However, in wet weight determination, oven-cured specimens were cured in water for 1 week, and then again, the weight gave initial wet weight (w_i,wet_). After determining the initial dry and wet weight, all the GPC specimens were placed in a chemical solution. The second weight (w_s,wet_) was specified on the day of testing by measuring the specimen weight just after cleaning the specimen. The second dry weight (w_s,dry_) was determined by considering the specimen after drying the specimen at room temperature.

#### 2.3.2. Water Absorption

The water absorption test was conducted on a 150 mm hard concrete cube in this study as per BS 1881: Part122, 1983 [49]. Initially, cubes were dried at 100 °C for 1 day, left at room temperature for 1 day, and then we measured the initial weight W1. After that, cubes were immersed in water and weighed at 1 h, 12 h, 24 h, 72 h intervals, giving the final weight W2. During this test, water was absorbed by GPC only through pores during immersion and discharged during drying in an oven. Water absorption is determined from Equation (1).

(1) $$Water\;Absorption\left(\%\right)=\frac{W2-W1}{W1}\times 100$$

#### 2.3.3. Sorptivity

The measure of the capability of GPC to percolate water through pores and transmit under capillary action is known as the sorptivity of concrete. Sorptivity is an important feature in studying the durability characteristics of GPC as it connects to the rate of liquid penetration into the concrete. In this study, sorptivity is determined before and after the chemical exposure to determine the influence of chemical exposure on the penetrating surface of the concrete. In order to perform the sorptivity test, a GPC cube of size 150 mm × 150 mm × 150 mm was prepared and dried in an oven for 1 day at a temperature of 60 °C and then kept left at room temperature for 1 day for cooling. After that, all the peripheral surfaces of the cube, including the top, were covered with tape except the base to protect from water entry from the sides and the top of the cube. The specimen was left in a container with water up to 3–5 mm from the base of the cubes. After 7 days, the specimens’ weight was measured to calculate the quantity of water absorbed. The bottom surface of the cube, exposed to chemicals, was wiped with a cloth before measuring the weight of the specimen. Sorptivity is then calculated with Equations (2) and (3) [50].

(2) $$S=\frac{I}{t^{1/2}}$$

(3) $$I=\frac{∆m_{t}}{a\times d}$$

where *t* is the time of exposure of the specimen surface to the liquid, $∆m_{t}$ is the variation in the mass of the specimen due to penetration of liquid, a is the area of the cube base in mm^2^, and d is the water density.

#### 2.3.4. Compressive Strength 

The compressive strength of GPC was examined on 150 mm × 150 mm × 150 mm cubes in accordance with IS: 516-1959 [51]. All four mix types were cast in moulds and then placed in an oven for 1 day at a temperature of 27 °C, 60 °C, 90 °C, and 120 °C and then left for 1 day for cooling at room temperature. Specimens were then placed in all four chemical solutions, i.e., sulphuric acid (2%), hydrochloric acid (2%), sodium chloride (6%), and sodium sulfate (6%), along with one tape water curing tank for comparison of strength loss due to chemical exposure. The compressive strength of GPC was examined after 28 days, 56 days, and 90 days of continuous immersion of the specimen in harmful chemicals.

## 3. Results and Discussion

### 3.1. Visual Appearance of Exposed GPC Specimens

GPC exposed to the chemical attack showed no deterioration during exposure to the solution of NaCl and solution of Na_2_SO_4_ but offered the formation of a 2 to 3 mm thickness white layer. This white layer is due to Na_2_CO_3_ formation after exposure to the solution of NaCl and Na_2_SO_4_. This white layer was observed after taking out the specimen from the chemical solution, cleaning the specimen with cloth, and exposing it to air, which is also observed in previous research [52,53]. The formation of Na_2_CO_3_ is due to the reaction of NaOH and CO_2_ in the atmosphere. It was observed that this sodium hydroxide leached out from the GPC after exposure to the chemicals [52]. The effect on the appearance of GPC is more by the HCl and H_2_SO_4_ as a comparison to the formation of GPC exposed to NaCl and Na_2_SO_4._

### 3.2. Density of GPC

**Figure 2:**
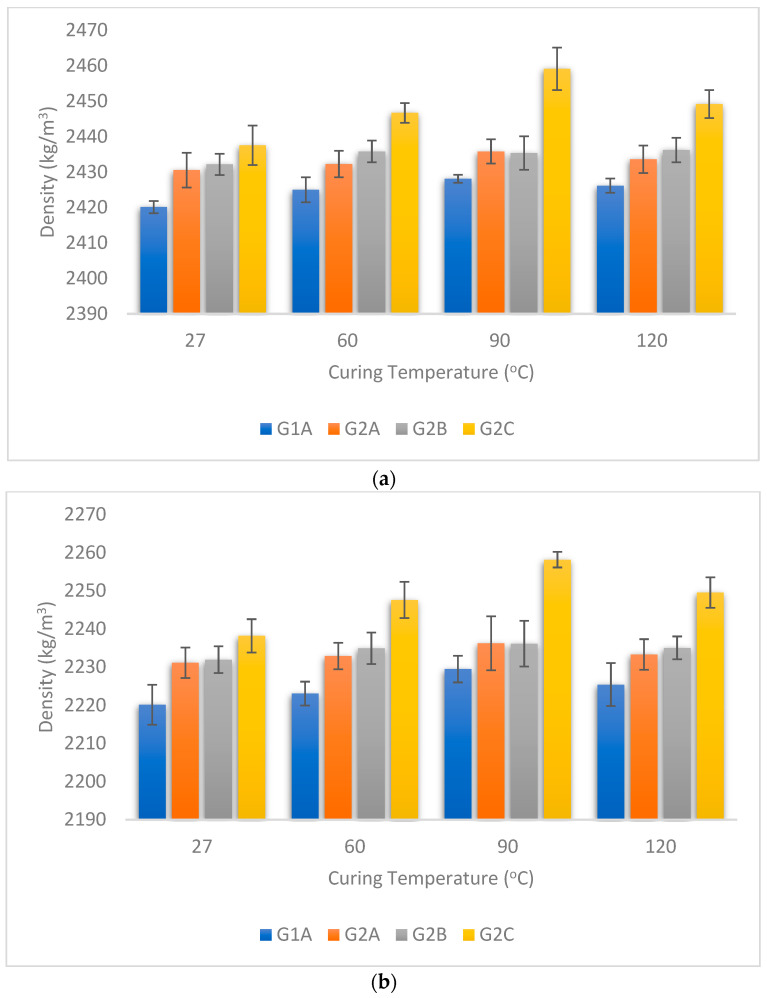

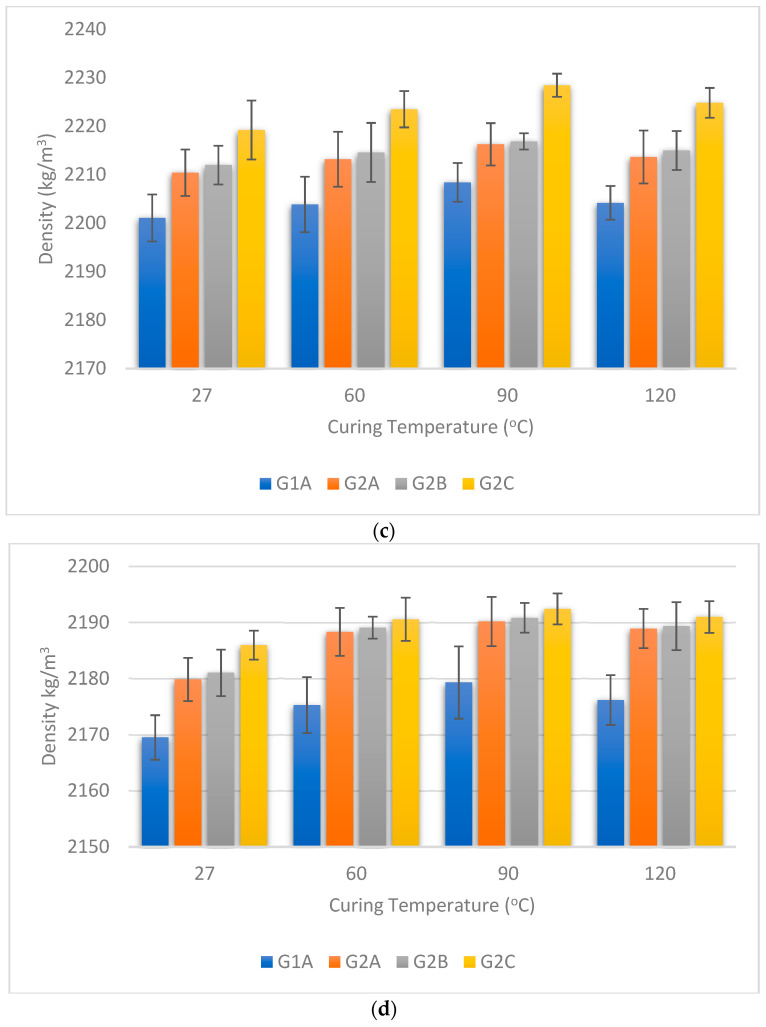

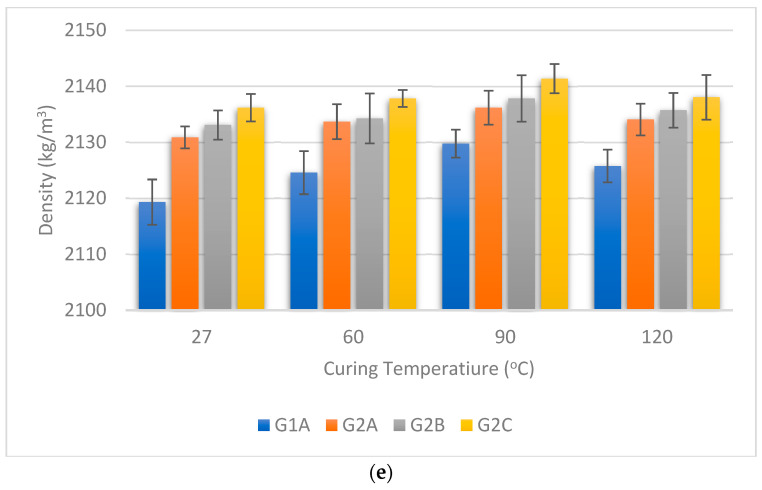
(**a**) Density of GPC cured under tap water for 90 days. (**b**) Density of GPC exposed to 6% NaCl for 90 days. (**c**) Density of GPC exposed to 6% Na_2_SO_4_ for 90 days. (**d**) Density of GPC exposed to 2% HCl for 90 days. (**e**) Density of GPC exposed to 2% H_2_SO_4_ for 90 days.

In this research, we studied the density of GPC when it was exposed to different conditions: tap water curing, 6% NaCl, 6% Na_2_SO_4_, 2% HCl, and 2% H_2_SO_4_ chemical solutions for 90 days. The results are shown in Figure 2a–e. In Figure 2a, we see that adding NS + SF combination generally increases the GPC density at all temperatures during tap water exposure. In addition, increasing curing temperature up to 90 °C boosts density, which then starts decreasing. Similarly, Figure 2b,c show a similar pattern for GPC exposed to NaCl and Na_2_SO_4_, respectively, with density increasing up to 90 °C and then decreasing. For Figure 2d, with 2% HCl exposure, the density rises with NS + SF at 27 °C but stays similar at higher temperatures. Finally, Figure 2e, displaying 2% H_2_SO_4_ exposure, shows density changes similar to 2% HCl exposure. Density generally increases with higher NS + SF percentages across various curing temperatures. Cevik et al. studied the impact of the temperature of curing on the change in mass after incorporation of NS (3%) in FA-based GPC, and it was observed that there is a gain in the mass of NS-incorporated GPC up to 1.3% exposed to chemical attack [41]. Similar results are observed by the other researcher in which an increase in the weight of GPC is observed after exposure to sulphate and chloride attack [54,55]. Law et al. studied the influence of the incorporation of NS on the density of GPC, and it was observed that nano-particles fill the pores of GPC and produce much denser GPC [56].

### 3.3. Water Absorption

**Figure 3:**
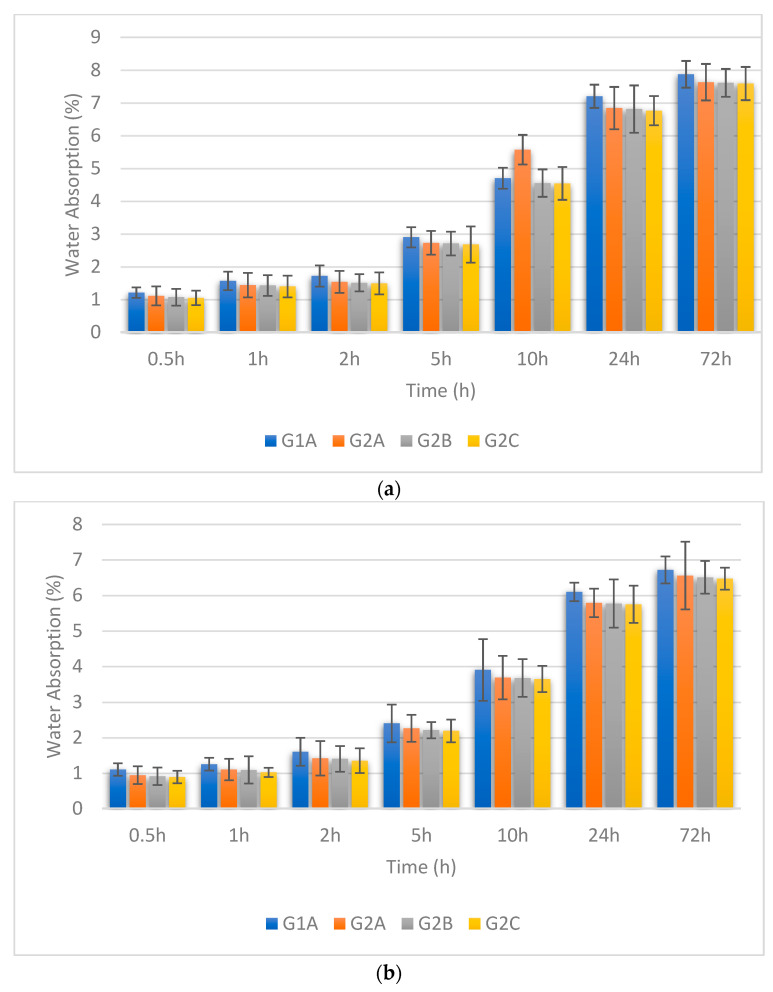

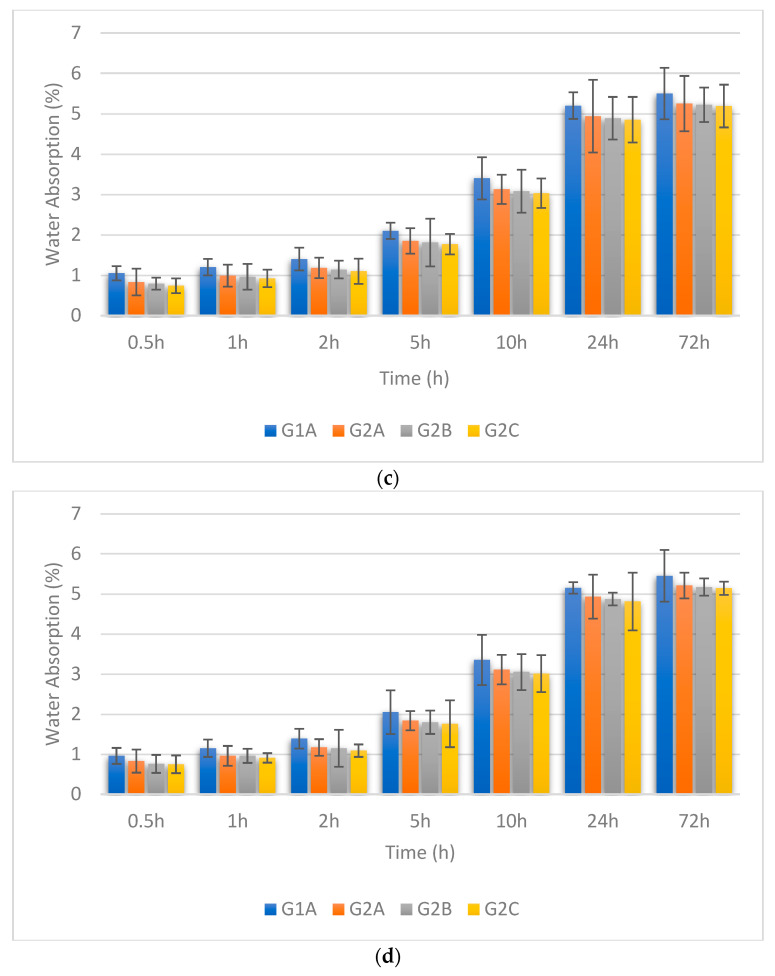
(**a**) Water absorption of different GPC mix cured at 27 °C. (**b**) Water absorption of different GPC mix cured at 60 °C. (**c**) Water absorption of different GPC mix cured at 90 °C. (**d**) Water absorption of different GPC mix cured at 120 °C.

In this study, we looked at water absorption in GPC with various treatments: curing at 120 °C, 90 °C, 60 °C, and 27 °C. In Figure 3a, for GPC cured at 27 °C, water absorption reduces as the NS + SF combination increases. Similar behaviour is seen in Figure 3b for GPC cured at 60 °C, where more NS + SF leads to less water absorption. This is because materials fill concrete gaps, making it less porous. Figure 3c,d reveal the same trend for GPC cured at 90 °C and 120 °C. Higher curing temperatures consistently result in lower water absorption. Gupta et al. studied the penetration of chloride ions and water absorption of GPC after incorporating a different percentage of SF, and it was noted that chloride ion penetration and water absorption are reduced by 26% and 23% after the incorporation of 10% SF in GPC which is similar to current research [57]. Nuaklong et al. studied the impact of the incorporation of NS on the water absorption of FA-based GPC in comparison with OPC using recycled aggregate. It was noted that water absorption is reduced after the incorporation of NS in GPC. However, during the incorporation of NS in GPC, it was noted that in NS-incorporated FA-based GPC, water absorption is increased with an increase in the percentage of NS up to 3% [58]. Jalal et al. studied the impact of the addition of NS and SF on the water absorption properties of self-compacting concrete (SCC), and it was observed that water absorption decreased by 35% and 31% after the incorporation of 2% NS and 10% SF, respectively [59]. Jalal et al. also studied the addition of 2% NS + 10% SF combination in SCC and observed that water absorption is decreased by 46% due to the addition of NS + SF combination compared with controlled GPC [59]. Adak et al. studied the impact of the incorporation of NS on water absorption of GPM undergoes ambient as well as heat curing, and it was noted that GPM cured at ambient temperature shows low water absorption and chloride penetration after incorporation of 6%NS in comparison with heat curing GPM [60].

### 3.4. Sorptivity

**Figure 4:**
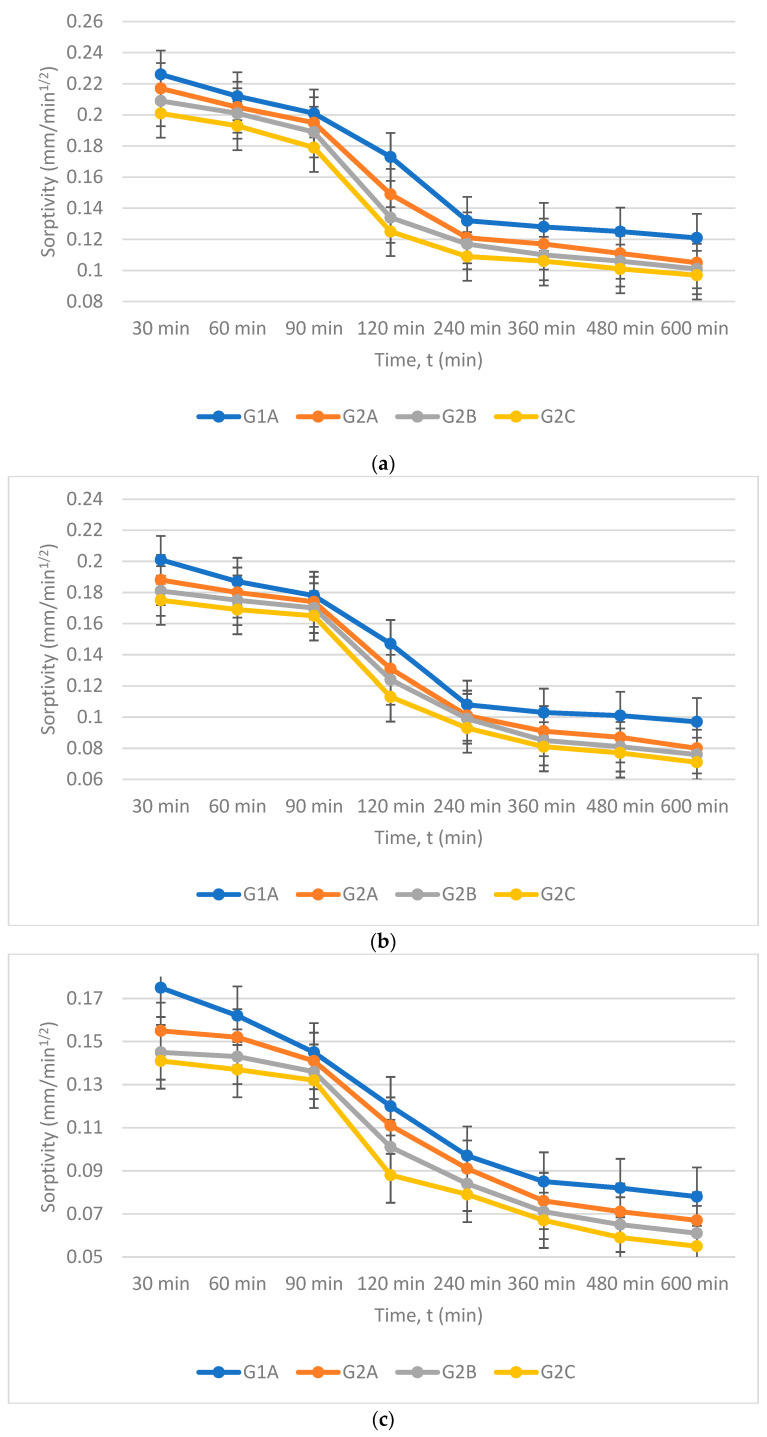

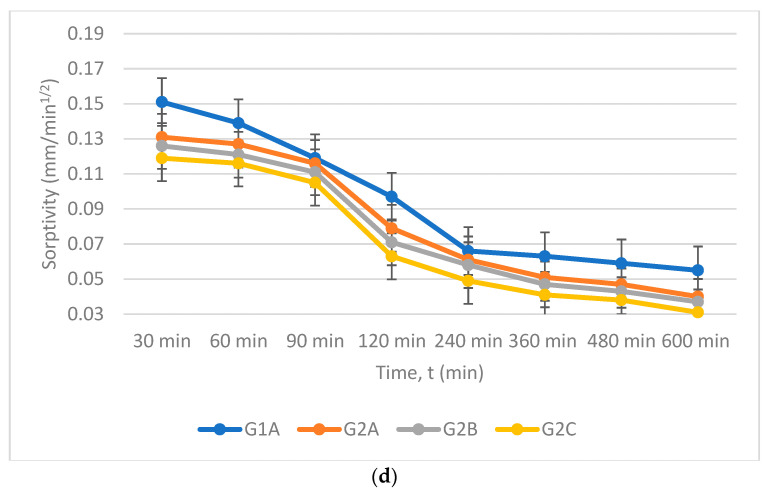
(**a**) Sorptivity of different GPC cured at 27 °C. (**b**) Sorptivity of different GPC cured at 60 °C. (**c**) Sorptivity of different GPC cured at 90 °C. (**d**) Sorptivity of different GPC cured at 120 °C.

The mechanism of movement of water within GPC cured at different temperatures is given by sorptivity, which is shown in Figure 4a–d. Secondary absorption is also controlled by reducing air voids after adding nano- and micro-silica, whereas initial absorption is due to the application of forces during capillary action. The sorptivity of different GPC cured at different temperatures is shown in Figure 4a–d. It is evident from the result that sorptivity is decreased with an increase in time. It was noted that sorptivity decreases after adding nano- and micro-silica in GPC for all curing temperatures. At an early age, the difference in sorptivity is not much for all GPC cured at different temperatures because absorption at an early age is due to capillary forces. The secondary age difference in sorptivity is very much due to the filling of pores by the NS and SF for all curing conditions.

Deb et al. studied the sorptivity of geopolymer mortar (GPM) after incorporation of NS in FA/GGBS-based GPM, and it was observed that the porosity of GPM is reduced just after incorporating 2% NS, which results in a decrease in sorptivity [61]. This decrease in sorptivity is due to the particle packing property of NS in the gaps of binder particles, an increase in the polymerisation rate and the production of a more aluminosilicate network. Their et al. studied the impact of adding NS and steel fibre in FA-based GPC. It was noted that the sorptivity of GPC is significantly decreased with an increase in the percentage of NS up to 2%. Still, the sorptivity of GPC is increased when we fix the NS percentage and increase the steel fibres percentage [62]. Saini et al. studied the impact of adding different percentages of NS in GGBS-based self-compacting GPC and noted that sorptivity is reduced with an increase in the percentage of NS [63].

### 3.5. Compressive Strength under Exposure to the Chemicals

**Figure 5:**
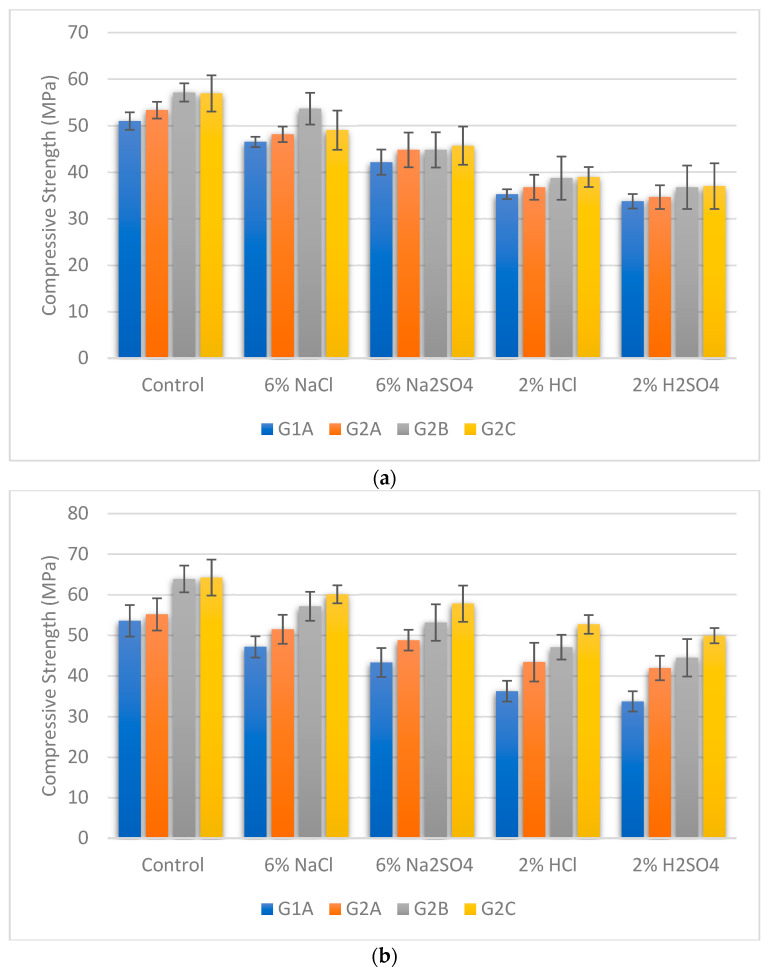

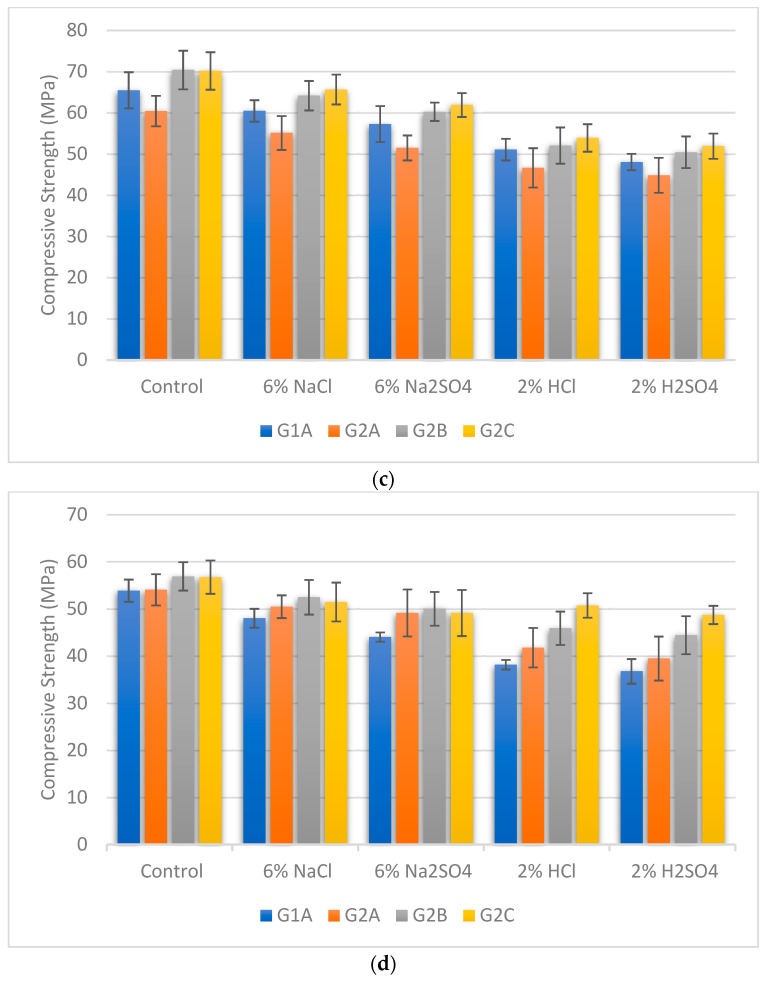
(**a**) Compressive strength of GPC cured at 27 °C exposed to chemicals for 90 days. (**b**) Compressive strength of GPC cured at 60 °C exposed to chemicals for 90 days. (**c**) Compressive strength of GPC cured at 90 °C exposed to chemicals for 90 days. (**d**) Compressive strength of GPC cured at 120 °C exposed to chemicals for 90 days.

During this study, we tested the strength of different GPC samples that were heated at different temperatures. We looked at how the strength changed after they were exposed to chemicals for 90 days. We also compared this with GPC samples that were just exposed to tap water for the same time. When GPC was cured at 27 °C and exposed to chemicals, its strength decreased, as shown in Figure 5a. The same kind of decrease was seen in GPC cured at 60 °C, as seen in Figure 5b. We also conducted similar tests for GPC cured at 90 °C and 120 °C, shown in Figure 5c,d. In all cases, the strength decreased when the GPC was exposed to chemicals. This decrease was more noticeable for acids such as HCl and H_2_SO_4_ compared to salts such as NaCl and Na_2_SO_4_. Among all the chemicals, H_2_SO_4_ had the most negative impact on GPC compressive strength. Bakharev et al. studied the effect of exposure of GPC to acidic media on the compressive strength of GPC, and it was observed that compressive strength is reduced after exposure to acidic media due to depolymerisation of aluminosilicate network [64]. The bridge of oxy-aluminium of the aluminosilicate network is destroyed in acidic media, resulting in compressive strength loss of GPC [65]. Many researchers studied the strength performance of FA/GGBS-based GPC in comparison with ordinary portland concrete (OPC) exposed to chemical attack, and it was observed that GPC has greater acid resistance [7,66]. Sothornchaiwit et al. studied the impact of the temperature of curing and the percentage of incorporation of SF on GPM compressive strength, and it was noted that the compressive strength of GPM exposed to sulphates is decreased with an increase in curing temperature [67]. A similar result was observed during the study of metakolin-based GPC after the incorporation of SF exposure to chemical attack, and it was noted that lowering of compressive strength due to chemical attack decreases with an increase in the percentage of SF [68]. 

## 4. Cost Analysis of GPC

In this section, we studied the cost of production of 1 m^3^ FA-based GPC for G40 having strength 40 MPa and compared it with the cost of production of 1 m^3^ OPC of M40 grade using IS, ACI, and DOE codes in Indian rupees (Rs). The quantity of material used in the production of GPC and OPC using different codes, along with their corresponding cost, are given in Table 4. The major ingredient of GPC is source material, which is the waste product of various industries, so its cost is negligible, including only transportation costs. The costlier ingredient of GPC is sodium hydroxide and sodium silicate, used as an alkaline activator in GPC. Hence, getting this material from waste stream material is important to minimise the production cost [69]. It is clearly shown in Table 4 that the cost of GPC is less than the cost of OPC designed using ACI and DOE codes, but the cost of GPC is a little more than that of OPC designed using IS codes. It is also noted from Table 4 that the maximum cost is obtained when we use the ACI method of designing OPC, and the minimum cost is obtained when we use the IS method for designing a mixed proportion of OPC. It was observed by many researchers that the cost of GPC is lesser than OPC due to the usage of waste material, and some researchers also observed that the cost of GPC is 1–2% higher than OPC [70,71]. Akhtar et al. studied the ecological footprint of GPC in comparison with OPC for M30 grade, and it was noted that the cost of concrete designed using DOE codes gives the minimum cost value, and the cost of GPC is less than the cost of OPC designed using IS codes [72].

## 5. Conclusions and Future Prospects

The outcome of replacing cement with supplementary material such as FA, GGBS, and AF in a fixed proportion using an alkaline activator reported in this paper can be summarised as follows:

- The addition of the NS + SF combination exhibited a consistent influence on the density of GPC specimens across different curing temperatures and chemical exposures. An upward trend in density was observed with the introduction of the NS + SF combination, indicating a potential to enhance the material’s compactness.
- The density response of GPC to different curing temperatures was notable. The density of GPC demonstrated an incremental trend with increasing curing temperature up to 90 °C, followed by a subsequent decrease. This indicates a complex interplay between temperature and material density.
- The density behaviour of GPC under various chemical exposures—NaCl, Na_2_SO_4_, HCl, and H_2_SO_4_—was consistent. Chemical attacks led to a reduction in density, indicating material deterioration. The magnitude of density decrease was observed to be in the range of 8.20% and 12.42% across the different curing temperatures.
- The reduction in water absorption, consistent across all NS + SF combinations at higher curing temperatures, accentuates the capacity of elevated curing temperatures to promote non-porous characteristics within the GPC matrix. Decline reflection in the water absorption as the curing temperature elevates to 90 °C and 120 °C, highlighting the consistent trend of improved moisture resistance.
- Quantifying the magnitude of impact, the data illustrates the percentage decrease in water absorption for various curing temperatures and NS + SF combination percentages. It is evident that higher NS + SF proportions and elevated curing temperatures yielded more pronounced reductions in water absorption percentages, reinforcing the moisture-resistant attributes of GPC.
- A noteworthy trend emerges with sorptivity decreasing over time. It is particularly striking that the impact of NS and SF additives consistently contributes to reducing sorptivity across all curing temperatures. It becomes evident that the effect of sorptivity reduction is more pronounced with increased curing duration.
- At an early age, the disparity in sorptivity across GPC specimens cured at varying temperatures is less pronounced due to the predominant influence of capillary forces. However, the divergence becomes more pronounced over time. The secondary age difference in sorptivity is attributed to the additive-driven filling of pores with NS and SF, a phenomenon consistent across different curing conditions.
- Adding NS and SF combined proportions contributes to enhanced compressive strength across all curing temperatures. Furthermore, it is evident that GPC’s compressive strength is more resilient against exposure to NaCl and Na_2_SO_4_ compared to the corrosive effects of acids (HCl and H_2_SO_4_). Among the chemicals tested, H_2_SO_4_ emerges as the most detrimental, leading to significant compressive strength deterioration.
- The role of NS and SF combined proportions in augmenting compressive strength across curing temperatures highlights the potential for improved structural integrity. These findings offer valuable insights into developing concrete structures that can effectively withstand chemical challenges, fostering the advancement of durable and sustainable construction practices.

## Figures and Tables

**Table 1:** Different types of oxides are present in FA, GGBS, SF, AF, and NS.

Sample (%)	SiO_2_	Fe_2_O_3_	CaO	Al_2_O_3_	MgO	K_2_O	Na_2_O	SO_3_	P_2_O_5_	TiO_2_	LOI ^a^
FA	49	12.5	2.79	27.25	0.89	0.46	0.32	0.38	0.98	1.54	0.64
GGBS	32.46	0.61	43.1	14.3	3.94	0.33	0.24	4.58	0.02	0.55	0.09
SF	85	1.5	0.8	1.1	2.5	0.9	1.3	1.3	-	-	2.8
AF	35.3	2.20	35.20	26.4	-	-	-	0.90	-	-	-
NS	92	-	0.70	-	-	0.25	-	0.80	-	-	2.42

^a^ = loss on ignition.

**Table 2:** The physical characteristics of FA, GGBS, AF, FiAg, and CoAg used.

Property	FA	GGBS	AF	SF	CoAg	FiAg
Specific gravity	2.41	3.12	2.89	2.20	2.89	2.61
Water Absorption (%)	-	-	-	-	0.98	1.35
Fineness Modulus	-	-	-	-	-	3.017
Specific surface area (m^2^/kg)	412	407	1200	-	-	-

**Table 3:** The specific values of constant parameters for each GPC mix (kg/m^3^).

Mix	FA	GGBS	AF	NaOH	Na_2_SiO_3_	SP	CoAg	FiAg
GPC	149.17	213.1	63.93	42.6	106.6	6	1276.8	547.8

**Table 4:** Cost Analysis of production of 1 m^3^ FA-based GPC (G40) and OPC (M40) using IS, ACI, and DOE codes.

S. No.	Material	Rate in Rs./Kg	GPC G40		IS M40		ACI M40		DOE M40	
			Quantity	Cost	Quantity	Cost	Quantity	Cost	Quantity	Cost
1	OPC	6.56	0	0	400	2624	356	2335.36	264	1731.84
2	Fly Ash	1.45	426.2	216.29	0	0	0	0	0	0
3	Coarse Aggregate	0.6	1276.8	766.08	1230	738	1170.8	1943.52	1359.8	2257.26
4	Fine Aggregate	1.4	547.8	766.92	410	574	714.8	1000.72	698.8	978.32
5	Sodium hydroxide	25.5	42.6	1086.3	0	0	0	0	0	0
6	Sodium Silicate	10	106.6	1066	0	0	0	0	0	0
7	Super Plasticizer	100	6	600	1.5	150	1	100	1.5	150
	Total cost (Rs.)			4501.59		4086		5379.6		5117.42

Note: A Rupees (abbreviated as Rs) is a unit in the Indian numbering system (1 USD equal 84 Rupees as on 27 May 2023).

## Data Availability

All data will be made available on request.

## References

[1] Wong L.S. (2022). Durability performance of geopolymer concrete: A review. Polymers.

[2] Kurtoglu A.E., Alzeebaree R., Aljumaili O., Nis A., Gulsan M.E., Humur G., Cevik A. (2018). Mechanical and durability properties of fly ash and slag based geopolymer concrete. Adv. Concr. Constr..

[3] Karthik A., Sudalaimani K., Vijayakumar C. (2017). Durability study on coal fly ash-blast furnace slag geopolymer concretes with bio-additives. Ceram. Int..

[4] Karakoc M.B., Türkmen İ., Maraş M.M., Kantarci F., Demirboğa R. (2016). Sulfate resistance of ferrochrome slag based geopolymer concrete. Ceram. Int..

[5] Paruthi S., Khan A.H., Kumar A., Kumar F., Hasan M.A., Magbool H.M., Manzar M.S. (2023). Sustainable cement replacement using waste eggshells: A review on mechanical properties of eggshell concrete and strength prediction using artificial neural network. Case Stud. Constr. Mater..

[6] Valencia Saavedra W.G., Angulo D.E., Mejía de Gutiérrez R. (2016). Fly ash slag geopolymer concrete: Resistance to sodium and magnesium sulfate attack. J. Mater. Civ. Eng..

[7] Okoye F.N., Prakash S., Singh N.B. (2017). Durability of fly ash based geopolymer concrete in the presence of silica fume. J. Clean. Prod..

[8] Paruthi S., Husain A., Alam P., Khan A.H., Hasan M.A., Magbool H.M. (2022). A review on material mix proportion and strength influence parameters of geopolymer concrete: Application of ANN model for GPC strength prediction. Constr. Build. Mater..

[9] Shi C., Jiménez A.F., Palomo A. (2011). New cements for the 21st century: The pursuit of an alternative to Portland cement. Cem. Concr. Res..

[10] Ahmed H.U., Mohammed A.A., Rafiq S., Mohammed A.S., Mosavi A., Sor N.H., Qaidi S.M. (2021). Compressive strength of sustainable geopolymer concrete composites: A state-of-the-art review. Sustainability.

[11] Kabir S., Alengaram U.J., Jumaat M.Z., Sharmin A., Islam A. (2015). Influence of molarity and chemical composition on the development of compressive strength in POFA based geopolymer mortar. Adv. Mater. Sci. Eng..

[12] Albitar M., Visintin P., Mohamed Ali M., Drechsler M. (2015). Assessing behaviour of fresh and hardened geopolymer concrete mixed with class-F fly ash. KSCE J. Civ. Eng..

[13] AL-Kharabsheh B.N., Moafak Arbili M., Majdi A., Ahmad J., Deifalla A.F., Hakamy A., Majed Alqawasmeh H. (2022). Feasibility study on concrete made with substitution of quarry dust: A review. Sustainability.

[14] Alraddadi S., Assaedi H. (2020). Characterization and potential applications of different powder volcanic ash. J. King Saud Univ. Sci..

[15] Zeyad A.M., Khan A.H., Tayeh B.A. (2020). Durability and strength characteristics of high-strength concrete incorporated with volcanic pumice powder and polypropylene fibers. J. Mater. Res. Technol..

[16] Topark-Ngarm P., Chindaprasirt P., Sata V. (2015). Setting time, strength, and bond of high-calcium fly ash geopolymer concrete. J. Mater. Civ. Eng..

[17] Nuruddin M.F., Demie S., Ahmed M.F., Shafiq N. (2011). Effect of superplasticizer and NaOH molarity on workability, compressive strength and microstructure properties of self-compacting geopolymer concrete. Int. J. Geol. Environ. Eng..

[18] Haustein E., Kuryłowicz-Cudowska A., Łuczkiewicz A., Fudala-Książek S., Cieślik B.M. (2022). Influence of cement replacement with sewage sludge ash (SSA) on the heat of hydration of cement mortar. Materials.

[19] Imtiaz L., Rehman S.K.U., Ali Memon S., Khizar Khan M., Faisal Javed M. (2020). A review of recent developments and advances in eco-friendly geopolymer concrete. Appl. Sci..

[20] Parathi S., Nagarajan P., Pallikkara S.A. (2021). Ecofriendly geopolymer concrete: A comprehensive review. Clean Technol. Environ. Policy.

[21] Allaoui D., Nadi M., Hattani F., Majdoubi H., Haddaji Y., Mansouri S., Oumam M., Hannache H., Manoun B. (2022). Eco-friendly geopolymer concrete based on metakaolin and ceramics sanitaryware wastes. Ceram. Int..

[22] Reddy D.V., Edouard J.-B., Sobhan K. (2013). Durability of fly ash–based geopolymer structural concrete in the marine environment. J. Mater. Civ. Eng..

[23] Pasupathy K., Sanjayan J., Rajeev P., Law D.W. (2021). The effect of chloride ingress in reinforced geopolymer concrete exposed in the marine environment. J. Build. Eng..

[24] Rajamane N., Nataraja M., Lakshmanan N., Dattatreya J. (2011). Rapid chloride permeability test on geopolymer and Portland cement. Indian Concr. J..

[25] Sathia R., Babu K.G., Santhanam M. (2008). Durability study of low calcium fly ash geopolymer concrete. Proceedings of the 3rd ACF International Conference-ACF/VCA, Ho Chi Minh City, Vietnam, 11–13 November 2008.

[26] Goriparthi M.R., TD G.R. (2017). Effect of fly ash and GGBS combination on mechanical and durability properties of GPC. Adv. Concr. Constr..

[27] Prusty J.K., Pradhan B. (2020). Effect of GGBS and chloride on compressive strength and corrosion performance of steel in fly ash-GGBS based geopolymer concrete. Mater. Today Proc..

[28] Okoye F., Durgaprasad J., Singh N. (2016). Effect of silica fume on the mechanical properties of fly ash based-geopolymer concrete. Ceram. Int..

[29] Memon F.A., Nuruddin M.F., Shafiq N. (2013). Effect of silica fume on the fresh and hardened properties of fly ash-based self-compacting geopolymer concrete. Int. J. Miner. Metall. Mater..

[30] Alanazi H., Yang M., Zhang D., Gao Z. (2017). Early strength and durability of metakaolin-based geopolymer concrete. Mag. Concr. Res..

[31] Esparham A. (2020). Factors influencing compressive strength of metakaolin-based geopolymer concrete. Modares Civ. Eng. J..

[32] Albidah A., Alghannam M., Abbas H., Almusallam T., Al-Salloum Y. (2021). Characteristics of metakaolin-based geopolymer concrete for different mix design parameters. J. Mater. Res. Technol..

[33] Jena S., Panigrahi R., Sahu P. (2019). Mechanical and durability properties of Fly ash Geopolymer concrete with silica fume. J. Inst. Eng. Ser. A.

[34] Assaedi H., Shaikh F., Low I.M. (2016). Characterizations of flax fabric reinforced nanoclay-geopolymer composites. Compos. Part B Eng..

[35] Assaedi H.S., Olawale M.D. (2022). Impact of nano-alumina on the mechanical characterization of PVA fibre-reinforced geopolymer composites. J. Taibah Univ. Sci..

[36] Garg R., Garg R., Eddy N.O., Khan M.A., Khan A.H., Alomayri T., Berwal P. (2023). Mechanical strength and durability analysis of mortars prepared with fly ash and nano-metakaolin. Case Stud. Constr. Mater..

[37] Tayeh B.A., Hakamy A., Amin M., Zeyad A.M., Agwa I.S. (2022). Effect of air agent on mechanical properties and microstructure of lightweight geopolymer concrete under high temperature. Case Stud. Constr. Mater..

[38] Thokchom S., Dutta D., Ghosh S. (2011). Effect of incorporating silica fume in fly ash geopolymers. Int. J. Civ. Environ. Eng..

[39] Saba A.M., Khan A.H., Akhtar M.N., Khan N.A., Koloor S.S.R., Petrů M., Radwan N. (2021). Strength and flexural behavior of steel fiber and silica fume incorporated self-compacting concrete. J. Mater. Res. Technol..

[40] Wani A.Y., Bhandari M. (2021). Effect of Ground Granulated Blast Furnace Slag, Silica Fume and Nano Silica on the Strength & Durability Properties of Concrete: A Contemporary Review. Proceedings of the IOP Conference Series: Earth and Environmental Science, Surakarta, Indonesia, 24–25 August 2021.

[41] Çevik A., Alzeebaree R., Humur G., Niş A., Gülşan M.E. (2018). Effect of nano-silica on the chemical durability and mechanical performance of fly ash based geopolymer concrete. Ceram. Int..

[42] Jaradat Y., Matalkah F. (2023). Effects of micro silica on the compressive strength and absorption characteristics of olive biomass ash-based geopolymer. Case Stud. Constr. Mater..

[43] Sikder A., Saha P. (2021). Effect of different types of Waste as Binder on Durability Properties of Geopolymer Concrete: A Review. Proceedings of the IOP Conference Series: Earth and Environmental Science, Surakarta, Indonesia, 24–25 August 2021.

[44] Bagchi S., Ghuleand S., Jadhav R. (2012). Fly ash fineness–Comparing residue on 45 micron sieve with Blaine’s surface area. IndIan ConCreTe J..

[45] (1987). Specification of Granulated Slag for the Manufacture of Portland Slag Cement.

[46] (2003). Specification for Silica Fume [CED 2: Cement and Concrete].

[47] (1970). Specification for Coarse and Fine Aggregates from Natural Sources for Concrete [CED 2: Cement and Concrete].

[48] Albitar M., Ali M.M., Visintin P., Drechsler M. (2015). Effect of granulated lead smelter slag on strength of fly ash-based geopolymer concrete. Constr. Build. Mater..

[49] Sivakumar V., Kavitha O., Arulraj G.P., Srisanthi V. (2017). An experimental study on combined effects of glass fiber and Metakaolin on the rheological, mechanical, and durability properties of self-compacting concrete. Appl. Clay Sci..

[50] Bellum R.R., Muniraj K., Madduru S.R.C. (2020). Influence of slag on mechanical and durability properties of fly ash-based geopolymer concrete. J. Korean Ceram. Soc..

[51] Siddique R. (2003). Effect of fine aggregate replacement with Class F fly ash on the abrasion resistance of concrete. Cem. Concr. Res..

[52] Bakharev T. (2005). Durability of geopolymer materials in sodium and magnesium sulfate solutions. Cem. Concr. Res..

[53] Singh N., Vyas S., Pathak R., Sharma P., Mahure N., Gupta S. (2013). Effect of aggressive chemical environment on durability of green geopolymer concrete. Int. J. Eng. Innov. Technol..

[54] Al-Dulaijan S., Macphee D., Maslehuddin M., Al-Zahrani M., Ali M. (2007). Performance of plain and blended cements exposed to high sulphate concentrations. Adv. Cem. Res..

[55] Thokchom S. (2014). Fly ash geopolymer pastes in sulphuric acid. Int. J. Eng. Innov. Res..

[56] Law D.W., Adam A.A., Molyneaux T.K., Patnaikuni I., Wardhono A. (2015). Long term durability properties of class F fly ash geopolymer concrete. Mater. Struct..

[57] Gupta A., Gupta N., Saxena K.K. (2021). Mechanical and durability characteristics assessment of geopolymer composite (Gpc) at varying silica fume content. J. Compos. Sci..

[58] Nuaklong P., Sata V., Wongsa A., Srinavin K., Chindaprasirt P. (2018). Recycled aggregate high calcium fly ash geopolymer concrete with inclusion of OPC and nano-SiO_2_. Constr. Build. Mater..

[59] Jalal M., Pouladkhan A., Harandi O.F., Jafari D. (2015). Comparative study on effects of Class F fly ash, nano silica and silica fume on properties of high performance self compacting concrete. Constr. Build. Mater..

[60] Adak D., Sarkar M., Mandal S. (2014). Effect of nano-silica on strength and durability of fly ash based geopolymer mortar. Constr. Build. Mater..

[61] Deb P.S., Sarker P.K., Barbhuiya S. (2016). Sorptivity and acid resistance of ambient-cured geopolymer mortars containing nano-silica. Cem. Concr. Compos..

[62] Their J.M., Özakça M. (2018). Developing geopolymer concrete by using cold-bonded fly ash aggregate, nano-silica, and steel fiber. Constr. Build. Mater..

[63] Saini G., Vattipalli U. (2020). Assessing properties of alkali activated GGBS based self-compacting geopolymer concrete using nano-silica. Case Stud. Constr. Mater..

[64] Bakharev T. (2005). Resistance of geopolymer materials to acid attack. Cem. Concr. Res..

[65] Chindaprasirt P., Rattanasak U., Taebuanhuad S. (2013). Resistance to acid and sulfate solutions of microwave-assisted high calcium fly ash geopolymer. Mater. Struct..

[66] Zhang P., Gao Z., Wang J., Guo J., Hu S., Ling Y. (2020). Properties of fresh and hardened fly ash/slag based geopolymer concrete: A review. J. Clean. Prod..

[67] Sothornchaiwit K., Dokduea W., Tangchirapat W., Keawsawasvong S., Thongchom C., Jaturapitakkul C. (2022). Influences of silica fume on compressive strength and chemical resistances of high calcium fly ash-based alkali-activated mortar. Sustainability.

[68] Aygörmez Y., Canpolat O. (2021). Long-term sulfuric and hydrochloric acid resistance of silica fume and colemanite waste reinforced metakaolin-based geopolymers. Rev. Constr..

[69] Abbas R., Khereby M.A., Ghorab H.Y., Elkhoshkhany N. (2020). Preparation of geopolymer concrete using Egyptian kaolin clay and the study of its environmental effects and economic cost. Clean Technol. Environ. Policy.

[70] Shaikh F. (2013). Deflection hardening behaviour of short fibre reinforced fly ash based geopolymer composites. Mater. Des..

[71] Thaarrini J., Dhivya S. (2016). Comparative study on the production cost of geopolymer and conventional concretes. Int. J. Civ. Eng. Res..

[72] Akhtar N., Ahmad T., Husain D., Majdi A., Alam M.T., Husain N., Wayal A.K.S. (2022). Ecological footprint and economic assessment of conventional and geopolymer concrete for sustainable construction. J. Clean. Prod..

